# Body image dissatisfaction and symptoms of depression disorder in adolescents

**DOI:** 10.1590/1414-431X202010397

**Published:** 2020-12-07

**Authors:** L.C. Soares, R.F.L. Batista, V.C. Cardoso, V.M.F. Simões, A.M. Santos, S.J.D.D.A.C. Coelho, A.A.M. Silva

**Affiliations:** 1 Departamento de Saúde Pública, Universidade Federal do Maranhão, São Luís, MA, Brasil; 2 Departamento de Puericultura e Pediatria, Faculdade de Medicina de Ribeirão Preto, Universidade de São Paulo, Ribeirão Preto, SP, Brasil; 3 Departamento de Medicina I, Universidade Federal do Maranhão, São Luís, MA, Brasil

**Keywords:** Mental disorders, Depression, Adolescent, Body dissatisfaction

## Abstract

The objective of this study was to evaluate the effect of body image dissatisfaction on symptoms of depressive disorder in adolescents. This is a cross-sectional study that included 2,162 adolescents ages 18-19 born in São Luís, Maranhão, Brazil, which was part of the joint RPS cohort (Brazilian birth cohorts of Ribeirão Preto-SP, Pelotas-RS, and São Luís-MA). Socioeconomic characteristics, nutritional status, mental health, and body image characteristics were evaluated. Body image was assessed by Stunkard’s silhouettes scale. The presence of symptoms indicative of depressive disorder was investigated through a diagnostic interview MINI (Mini International Neuropsychiatric Interview). A theoretical model was built in a Directed Acyclic Graph (DAG) in order to investigate the relationship between the variables of the study. The relationship was estimated weighting the inverse probability of selection for the variables of adjustment: sex and nutritional status. Among the dissatisfied adolescents due to overweight, 66.54% were girls, 32.85% were overweight, and 11.99% were obese (P<0.01). There was a significant association between dissatisfaction due to overweight and symptoms of depressive disorder (P=0.01), and there was no evidence of the same association with dissatisfaction due to thinness. Therefore, only dissatisfaction due to overweight was associated with the symptoms of depressive disorder in the evaluated adolescents.

## Introduction

Evidence suggests a possible bidirectional association between depression and body image. Perhaps, either depression worsens body image perception or a distorted perception of body image favors depression (1). In this sense, body image can be defined as a multidimensional construction that describes the internal representations of the body structure and physical appearance of individuals in relation to themselves and others (2).

During adolescence, there are high concerns with physical appearance regarding body shape, especially when referring to overweight (3). In line with this, discriminatory attitudes towards young people whose physical shape is not compatible with the imposed standards, namely a thin body for girls and muscular and tall body for boys, are related to body image dissatisfaction (BID) (4), characterized as a negative evaluation towards a person's own physical appearance through feelings of discomfort (5).

Scientific literature indicates that BID is closely related to eating disorders, such as anorexia and bulimia nervosa (6). On the other hand, other studies attempt to elucidate the relationship of BID with other types of psychological variables, such as depression (7,8). Thus, the association between body image and depressive behaviors is not completely clear, and it is not known if the concerns with physical appearance cause depression or if individuals with depression are more susceptible to have BID (9).

Regarding psychological variables, depressive disorder is a severe psychiatric illness characterized by depressive mood and decreased interest or pleasure in performing daily activities (10). This illness represents one of the main causes of disability in the world, affecting nearly 300 million people of all ages (11). Its prevalence in individuals from the ages of 18 to 29 is three times higher than in people over 60 years old (10). Depression increases significantly during adolescence, being lower during childhood and gradually increasing until the age of 19, usually more intensely in girls (12). Considering that adolescence is a critical period for construction and establishment of body image perception, it has become increasingly important to understand more about the effects that BID may have on psychological aspects during this stage of life (13).

Based on the findings of Paans et al. (1), the majority of research that tried to elucidate the association between depression and body image focused on adolescents, suggesting associations between symptoms of depressive disorder and aspects related to body image perception. In this context, the objective of the present study was to investigate if BID affects depressive disorder in adolescents at ages 18 and 19, the period that is defined as the end of adolescence according to the World Health Organization (WHO) (14).

Therefore, the relevance of the present study is that it verified such effect in a large sample of adolescents, which was larger than that of important studies that cover a similar theme (7–9,15). Moreover, it used validated instruments for the investigation of symptoms that are indicative of depressive disorders, such as the Mini International Neuropsychiatric Interview (MINI), characterized as a standardized fast application tool that explores the main psychiatric disorders from the Diagnostic and Statistical Manual of Mental Disorders IV (16). Furthermore, this study tested the hypothesis that BID affects depressive disorders in adolescents and proposed to contribute to a greater understanding about this possible association.

## Material and Methods

This was a cross-sectional study derived from a cohort study with individuals that were born in São Luís, Maranhão, Brazil. The cohort is included in a joint cohort (RPS cohort, Brazilian birth cohorts of Ribeirão Preto-SP, Pelotas-RS, and São Luís-MA) developed by the Federal University of Maranhão (Universidade Federal do Maranhão-UFMA), Ribeirão Preto Medical School (Faculdade de Medicina de Ribeirão Preto-USP), and Federal University of Pelotas (Universidade Federal de Pelotas-UFPel). Only data from São Luís-MA was used in this study.

The participants of the RPS cohorts were evaluated in three phases. The first phase started at birth, from March of 1997 to February of 1998, including 96.3% of births during that period through systematic sampling with proportional stratification, according to the number of births in each maternity hospital, with one out of every seven births being selected, giving a total of 2,832 births. Non-residents of São Luís, twins, and stillbirths were excluded. The final sample was 2,443 births, with a 5.8% loss due to refusals or early discharge from the hospital (17). The second phase occurred when the children were 7-9 years old in 2005-2006 (18).

For the follow-up in the third phase, in 2015, the participants were invited to return for a new evaluation at ages 18 and 19. Data from the third phase were used in this article. The adolescents were identified through a search in the compulsory Military Conscription records in São Luís, in schools through the 2014 school census, and in universities, producing a total of 684 participants. Results regarding nutrition, mental health, and human capital were included in the evaluation (19).

With the objective of increasing the sample power, new individuals that were born in São Luís in 1997 were included. The first search was a drawing using SINASC database (Brazilian Live Births Information System). Being born in a maternity hospital in São Luís in 1997 was a criterion that was taken into consideration for the registration in the database. From these lists, a new random drawing was performed and resulted in a total of 4,593 individuals. From the total, phone or in-person contact was conducted with 1,133. In the second stage, 695 more volunteers that were born in the same year were identified in schools, universities, and through social media, giving a total of 1,828 adolescents. The members of the cohort and the rest of the participants were all submitted to the same tests and surveys. The total sample was 2,515 participants from São Luís (19). For this article, the final sample resulted in 2,162 adolescents, due to losses of mental health and body image information.

Data collection was conducted at UFMA. Health professionals were hired and trained for the conduction of research surveys and handling of equipment (19). The following variables and instruments were used:

###  

#### Socioeconomic characteristics

Sex (male/female); economic class from the Brazilian Criteria for Economic Classification 2016 (A, B1, B2, C1, C2, D/E – class A being the richest and with the highest levels of education, and classes D/E, the poorest and with the lowest levels of education – Brazilian Association of Research Companies <http://www.abep.org/criterio-brasil>); currently attends school/college (yes/no); currently works (yes/no); neither works nor attends school/college (yes/no).

###  

#### Humiliation from jokes about body shape

Never/ rarely/ sometimes/ almost always/ always, using a structured survey.

###  

#### Nutritional status

Body mass was measured by a Filizola^®^ scale attached to the BodPod^®^ instrument, and height was measured by Altura Exata^®^ stadiometer (all from Brazil). The WHO's growth curves, proposed in 2007, were adopted for the nutritional status classification according to the BMI-for-age and reference for sex in score-z. Therefore, the participants with BMI-for-age with score-z <-2 are classified as low weight, those with scores-z ≥-2 and <+1 are classified as normal weight, ≥+1 and <+2, overweight, and score-z ≥+2, obese (20 [Bibr B21]).

###  

#### Mental health

Current or recurrent major depressive episode (present/absent) and major depressive episode with melancholic features (present/absent) using the MINI questionnaire (Brazilian version 5.0.0 - DSM IV). After the investigation of the episodes, the dichotomous dependent variable “Presence of Depressive Disorder symptoms” was created considering the presence of at least one of the referred episodes. All participants that presented any mental health problem identified through MINI were referred to outpatient specialized follow-up care (19).

###  

#### Body image

Data was collected by the use of the silhouettes scale proposed by Stunkard et al. (22), previously validated for the Brazilian population (23,24). This scale is composed of a group of human figures numbered from 1 (thinnest) to 9 (the most obese). The scale was presented to the adolescents, and they answered two questions: Which one of these figures do you identify your body with the most? Which one of these figures looks like what you'd like your body to be? Each adolescent picked the number of the silhouette that they considered the most similar to their image (real image), and the one they would like it to be (ideal image). BID was defined as the perceived body size minus the perceived ideal body size (1,25). When this difference resulted in zero, the individuals were classified as satisfied with their body image; when positive, they were considered dissatisfied due to overweight (desire to reduce body silhouette); when negative, dissatisfied due to thinness (desire to increase the silhouette) (26).

A descriptive analysis of the variables evaluated in the study was conducted. The categorical variables are presented in absolute frequencies and percentages. The difference between proportions according to the body image categorization (satisfied, dissatisfied due to overweight, and dissatisfied due to thinness) was performed by the chi-squared test. The fixed significance level was 5% and the confidence interval, 95% (95%CI). The program DAGitty (dagitty.net/dags.html) was used to create a theoretical model in a directed acyclic graph (DAG). The variables used in model adjustment according to the back-door criterion were nutritional status and sex. The estimate of the relationship was performed using inverse probability of weighting in marginal structural model using the variables selected from the DAG. A balanced design was conducted through the absolute standardized differences (<0.25) of the predictor variables among the groups of exposed (dissatisfied due to overweight or thinness) and non-exposed (satisfied) participants, and of the variance ratios, between 0.80 and 1.20. The variables with P<0.05 were considered significant. Data were analyzed using the statistics software STATA^®^ version 14.0 (https://www.stata.com).

The study met the criteria of the Resolution 466/2012 from the National Board of Health and Operational Norm 001/2013 CNS. The adolescents that agreed to participate in the research signed the informed consent form. The project and consent were approved by the Committee of Ethics in Research from the University Hospital (Consolidated Opinion number 1,302,489; October 29, 2015).

## Results

A total of 2,162 adolescents between 18 and 19 years old were evaluated. The results are presented in body image categories: satisfied, dissatisfied due to overweight, and dissatisfied due to thinness. Dissatisfaction due to overweight was more prevalent in girls (66.54%), and dissatisfaction due to thinness was greater in boys (59.76%), presenting a statistical difference. Regarding socioeconomic characteristics, the majority of the adolescents belonged to economic class C, especially those that were dissatisfied due to thinness (51.82%). On the other hand, in class A (4.77%) and B (26.64%), there was a greater percentage of adolescents that were dissatisfied due to overweight. In relation to occupation, the majority of the adolescents that neither worked nor attended school/college were dissatisfied due to thinness (25.69%), whereas 76.69% of those that were dissatisfied due to overweight had some kind of occupation, either worked or attended school/college (Supplementary Table S1).

Statistically significant differences were found in the condition of suffering humiliation from jokes about body shape, nutritional status, and presence of symptoms of depressive disorder between the satisfied and dissatisfied adolescents. It was verified that the conditions of suffering humiliation “almost always” (4.66%) and “always” (2.22%) were more present in those that were dissatisfied due to overweight, as well as the condition of “never” having suffered humiliation (73.45%) was more prevalent in those that were more satisfied with their body image. Regarding nutritional status, 90.52% of those dissatisfied due to thinness and 55.16% of the ones dissatisfied due to overweight, although dissatisfied, had adequate weight. However, among the adolescents that were classified with overweight and obesity, there was higher prevalence of dissatisfaction due to overweight (32.85 and 11.99%, respectively) compared to those that had adequate weight and those with low weight. In relation to the presence of symptoms of depressive disorder, the highest prevalence was verified among the adolescents that were dissatisfied due to overweight (17.20%), followed by those dissatisfied due to thinness (12.35%). Among the participants in which symptoms of depressive disorder were absent, a higher prevalence of satisfaction with body image was observed (88.42%), i.e., depression symptoms were observed mainly in the dissatisfied adolescents (Supplementary Table S1).

Table 1 shows the balancing between groups of satisfied and dissatisfied adolescents with their body image due to overweight and thinness, having interchangeability among the variables.

**Table 1:** Balancing of the variables by weighting with sample propensity score.

Variables	Dissatisfied due to overweight and symptoms of depressive disorder				Dissatisfied due to thinness and symptoms of depressive disorder			
	Standardized differences*		Variance ratios**		Standardized differences*		Variance ratios**	
	Gross value	Weighted value	Gross value	Weighted value	Gross value	Weighted value	Gross value	Weighted value
Nutritional status***	1.07	0.10	8.61	1.10	0.07	-0.03	0.73	0.48
Sex	0.32	0.14	0.88	0.95	-0.20	0.00	0.96	1.00

Data are reported as standardized differences and variance ratios, and gross and weighted values. Data are from only São Luís, Maranhão of the RPS cohort (joint Brazilian birth cohorts Ribeirão Preto, Pelotas, and São Luís) by classification of body image. *Values close to 0 (zero) indicate similarity between the groups. **Values close to 1 (one) indicate similarity between the groups. ***Based on BMI-for-age (body mass index, kg/m^2^) 20.

Table 2 shows the analysis of the effect of BID in depressive disorder in adolescents. There was a statistically significant association between dissatisfaction due to overweight and symptoms of depressive disorder (P=0.01). However, there was no significant association between dissatisfaction due to thinness and symptoms of depressive disorder (P=0.339) among the participants.

**Table 2:** Effect of dissatisfaction with body image on depressive disorders in the evaluated adolescents, weighted by the inverse probability of selection.

Symptoms of depressive disorder	Coefficient	Standard error	Z	P value	95%CI
Satisfied *vs* Dissatisfied due to overweight	0.05	0.02	2.56	0.01	0.013; 0.105
Satisfied *vs* Dissatisfied due to thinness	0.01	0.01	0.96	0.339	−0.019; 0.056

Data are from only São Luís, Maranhão of the RPS cohort (joint Brazilian birth cohorts Ribeirão Preto, Pelotas, and São Luís) by classification of body image.

95%CI: confidence interval of 95%.

## Discussion

Based on the results of the present study, BID was associated with symptoms of depressive disorder in the evaluated adolescents. In this context, when categorizing adolescents that are dissatisfied with overweight and thinness and when evaluating such effect, adjusting for nutritional status and sex, the effect on depressive disorder symptoms was revealed only in those that were dissatisfied due to overweight.

The results suggest that girls were more affected by this aspect, since they were more prevalent in the dissatisfied due to overweight category, and boys were more dissatisfied due to thinness. This could be a reflex of different vulnerability by sex (27) to the patterns imposed by media and cultural ideals (28) of higher appreciation of a thin female body and a muscular male body (27). Chen et al. (29) showed that female adolescents have a higher level of BID. Due to strong social pressure, girls tend to give more value to physical appearance, and the association between BID and psychological problems is stronger in groups that value physical appearance more. Therefore, it would be expected that body image has a higher effect in depression among these groups. As a result of perception of body changes that occur during adolescence, girls, mainly before the age of 19, could become more vulnerable to negative perceptions of body image as they are exposed to the current ideal of beauty (27), when noticing that such patterns do not correspond to their real body shape (28). The “ideal thin body” is difficult to achieve, thus leading mainly girls to frustrating efforts to change their body shape through, for example, restrictive diets. This can contribute to depression due to the emotional suffering that comes with several failed attempts (30). In boys, usually there is more effort to increase muscle mass through physical activity, and dietary restrictions as a result of BID are less common (30). However, a few studies have already revealed that the concern with overweight in men is increasingly more prevalent (31), and that BID could vary according to race, ethnicity, and sexual orientation in men (7).

Among the adolescents in the dissatisfied due to overweight category, there was higher prevalence of overweight and obesity compared to the satisfied ones and dissatisfied due to thinness. Langoni et al. (15), when evaluating the factors associated to body image in adolescents, verified that young people with overweight and those with adequate weight presented 2.0 and 5.2 more BID compared to those with low weight. Thus, the findings of the present study may be explained by the fact that adolescents with excess body mass present more chances of being socially marginalized (4). They could be more exposed to bullying due to the characteristics that differentiate them from others, especially due to overweight (27). Depressive symptoms could be more associated with body image distortion among those (4).

BID increases with overweight and obesity, but also affects the adolescents that have adequate weight (24). In the present study, in the adolescents with adequate weight, there was a high prevalence of BID, especially related to dissatisfaction due to thinness, which contrasts with other findings (31). This means that, although these adolescents have adequate weight in relation to their height, they still see themselves with low weight. Therefore, BID could be more associated with psychological harm than the alterations of body composition. Attention should be given not only to adolescents with inadequate nutritional status, but also to those that do not have a good perception of their own body (32). Blashill and Wilhelm (7), in a longitudinal study with adolescents and young adults, verified that boys that had adequate weight but perceived themselves with low weight, presented higher levels of depressive symptoms. Beyon et al. (33) observed that girls that perceived themselves with overweight but had adequate weight were more prone to depressive symptoms compared to those that had a more precise perception of their own weight.

Paans et al. (1) demonstrated that depression and higher values of BMI contribute independently to a higher BID. Thus, there is a significant need for treatment to reduce BID in those that suffer from depression and overweight. BID can have long-lasting consequences, such as the development of eating disorders like anorexia and bulimia nervosa, besides the adoption of unhealthy practices and lifestyles (1), such as inadequate compensatory methods of weight control, like self-induced vomiting (34), restrictive diets, exaggerated practice of physical activities, abusive use of laxatives and diuretics, anabolic steroids, and unnecessary plastic surgeries (27,35).

Flores-Cornejo et al. (8), when evaluating the association between BID and depressive symptoms in adolescents between 11 and 17 years old, demonstrated that those that had BID were 3.7 times more prone to reporting depressive symptoms, which could be measure by the presence of bullying and chronic stress, a mechanism that is still not explained. Feelings of discrimination, loneliness, sadness, and suicide planning are a few of the factors that have already been proven to be related to BID in adolescents, especially in individuals with overweight and obesity, which are more affected by self-esteem problems and depression (15).

Humiliation from jokes about body image in the present study were more prevalent in the participants with BID. The use of jokes or nicknames among these adolescents is very frequent, and serves as a way to emphasize the negative characteristics that they would like to hide in their bodies, creating prejudice and, consequently, depression. The imposed negative influence of body image perception lead adolescents to be slaves of beauty patterns and lose themselves in the search for their own identities in order to avoid feeling excluded from their social circles (4).

Although having a cross-sectional design, this study was nested in a cohort study and presents limitations that are common to that design, such as the lack of representability due to the difficulties in finding the adolescents and sample loss due to non-collected data on exposure and outcome variables. Therefore, it was necessary to incorporate a retrospective cohort (19). Another limitation is related to the reliability of the responses of the adolescents regarding the silhouette scale. It is not possible to assure that they chose the true figure or that maybe the selection was influenced by embarrassment or shyness, although they were well guided by the trained interviewer and the interview took place in a reserved place.

Among the strengths of the study, it is important to highlight the large sample of participants with similar age, 18 to 19 years old, in the final stages of adolescence according to the definition proposed by the WHO and followed by the Ministry of Health (14). Therefore, the effect of BID in symptoms of depressive disorder could be observed specifically at this stage, which differentiates this study from other works that evaluated samples of adolescents with greater age range (8,15,24,28,31,32). In this context, Simões et al. (19), when estimating the prevalence of health indicators in adolescents between the ages of 18 and 19 in São Luís, found a high prevalence of psychiatric disorders, emphasizing that there are important risk factors that increase the vulnerability of adolescents to mental disorders, and that the early exposure to these factors lead to development of diseases, increasing future morbidity and mortality.

### Conclusions

In this study, it was possible to conclude that BID was associated with symptoms of depressive disorder only in the adolescents dissatisfied with their body image due to overweight, and not in those dissatisfied due to thinness, adjusted for nutritional status and sex. It is important to emphasize that a higher prevalence of adolescents with overweight and obesity were in the dissatisfied due to overweight category. This category also contained a high prevalence of adolescents with adequate weight but that perceived themselves as overweight, consequently being dissatisfied. Therefore, healthcare actions and public policies directed towards prevention and treatment of depression in adolescents with BID must involve all adolescents, regardless of nutritional status.

## Supplementary Material

Click here to view [pdf].
